# Electrospinning over Solvent Casting: Tuning of Mechanical Properties of Membranes

**DOI:** 10.1038/s41598-018-23378-3

**Published:** 2018-03-22

**Authors:** Kajal Ghosal, Aniruddha Chandra, Praveen G., Snigdha S., Sudeep Roy, Christian Agatemor, Sabu Thomas, Ivo Provaznik

**Affiliations:** 1 Center for Nanoscience and Nanotechnology, Mahatma Gandhi University, Priyadarshini Hill, Kottayam, 686560 Kerala India; 2 Electronics and Communication Engineering Department, National Institute of Technology, Durgapur, 713209 West Bengal India; 3 Department of Biomedical Engineering, Faculty of Electrical Engineering and Communication, Brno University of Technology, Brno, Czech Republic; 4 School of Engineering and Applied Sciences, Harvard University, 52 Oxford Street, Cambridge, MA 02138 USA; 5 Department of Physiology, Faculty of Medicine, Masaryk University, Brno, Czech Republic

## Abstract

We put forth our opinion regarding the enhanced plasticity and modulation of mechanical properties of polymeric films obtained through electrospinning process in this article. In majority of the pharmaceutical, biomedical, and packaging applications, it is desirable that polymer based matrices should be soft, flexible, and have a moderate toughness. In order to convert inflexible and brittle polymers, adjuvants in the form of plasticizers are added to improve the flexibility and smoothness of solvent casted polymer films. However, many of these plasticizers are under scrutiny for their toxic effects and environmental hazards. In addition, plasticizers also increase the cost of end products. This has motivated the scientific community to investigate alternate approaches. The changes imparted in membrane casted by electrospinning were tried to be proved by SEM, Mechanical property study, DSC and XRD studies. We have showed dramatic improvement in flexibility of poly(ε-caprolactone) based nanofiber matrix prepared by electrospinning method whereas solvent casting method without any plasticizer produced very brittle, inflexible film of PCL. Modulation capacity of mechanical properties is also recorded. We tried to support our opinion by citing several similar findings available in the open literature. The electrospinning method helps in plasticization and in tuning mechanical properties.

## Introduction

Polymers, be it synthetic, semi-synthetic, or natural, have gained wide attention in different technology sectors. They are used in various forms, such as solid films, thin matrices, nanofiber mats or patches, hydrogel films etc.^1,2^. All these polymer forms are obtained from their original bulk form after appropriate modification of mechanical properties^3,4^. Further modification is often needed depending on the target application. One such important modification, especially for pharmaceutical, biomedical, and packaging industries, is to increase the flexibility of the polymer films through incorporation of plasticizer. As the plasticity of a polymer film turns out to be one of the most crucial fabrication points, similarly mechanical properties of films should also be considered during different applications. Polymeric films that are widely used as coatings over solid pharmaceutical dosage forms to improve their appearance and mechanical strength, to prevent them from dusting, chipping, and cracking, and to modify release of active ingredients from formulations are taken care of. In their basic form, these film coatings are often hard, brittle, and have lower ductility. Proper adjuvants are added during the manufacturing process to overcome these limitations.

Several investigators have confirmed the utility of polymeric film as a drug delivery carrier to avoid hepatic first pass metabolism and to reduce side effects related with other dosage forms. Systems that involve drug administration through adhesive bases, e.g., transdermal systems, bioadhesive vaginal films, sublingual or buccal films, wound dressing films, are all primarily comprised of polymer films that contain drugs. Further, using films as carrier attains some other goals such as enhancing bioavailability, realizing controlled/sustained and/or targeted drug release, minimizing drug dosage, and elevating patient compliance. Of course all the goals are accomplished only when conformability criterion for these thin films is compiled during formulation, i.e. they are strong and flexible enough for practical use.

Polymeric films have gained attention of the biomedical community as well^5^. Biomedical device construction is challenging because it is difficult to fabricate devices that are both biocompatible and portable^6^. Moreover, majority of the conventional biomedical devices lack flexibility which is of serious concern if they are to be worn or implanted to the patient body. The rigidity can be overcome with biopolymer based matrix or thin films. The films are biodegradable and this aspect is an added advantage.

Polymer films, either in membrane or in matrix forms, can contribute hugely in packaging, optical lenses, touch screens, foldable sensors, and each of these domains has their own set of plasticity and mechanical property requirements. In pharmaceutical, biomedical, and packaging industries there is a demand for flexible films. This requirement is fulfilled through incorporation of polymer specific plasticizers such as phtalates, trimellitates, adipates, sebacates, glycols, polyethers and alkyl citrates. In fact for the mentioned target applications plasticizers have become a very important additive in polymer systems, and modification of plasticity with plasticizer forms an integral part during fabrication irrespective of the film fabrication or matrix formation process. Table 1 summarizes how the plasticity of several solvent-cast polymer films is modified with the introduction of plasticizer. The cells (in Table 1) with no entries represent values that are either not calculated or could not be determined due to very brittle nature of film^7–20^.

**Table 1:** Effect of plasticizer on flexibility of different polymer films.

Polymer	Plasticizer	without Plasticizer				with Plasticizer			Application
		UTS (MPa)	% Elongation at break		Young’s Modulus (MPa)	UTS (MPa)	% Elongation at break	Young’s Modulus (MPa)	
PVA^7^	Sorbitol	14.36	61.23		75.9	5.36	110.81	22.6	Vaginal film
Eudragit RS PO^8^	Citric Acid	4.46	—		760	1.00	117.11	520	Controlled drug release film
HPMC^9^	TEC	83.3	8.5		891.1	76.7	107.0	516.9	Transdermal drug delivery
Chitosan and Carbopol^10^	Glycerol	4.56	44		—	4.5	65	—	Transdermal drug delivery
Zein^11^	Glycerol	—	—		—	24	0.029	1480	Drug release film
PMVE/MA^12^	Glycerol	18.23	1.68		24.07	4.73	14.72	1.14	Bioadhesive patch
PMVE/MA^13^	PEG 10000	—	—		—	31.02	478.43	9.32	Bioadhesive film
PVP-PEGDA-PEG^14^	PG	0.135	42.5		—	0.09	75	—	Pressure sensitive adhesive
HPC and EC^15^	PG	10.51	15.51		70.06	7.82	26.62	30.07	Buccal film
Soluplus^16^	TEC	8.5	2.6		472.4	5.1	178.5	70.0	Drug release film
	PPG	13.4	25.9		326.2	6.8	119.6	121.1	
	Glycerin	10.5	47.3		279.1	4.7	195.5	77.5	
**Polymer**	**Plasticizer**	**Plasticizer at low level**				**Plasticizer at high level**			**Application**
		**UTS** **(MPa)**	**% Elongation** **at break**	**Young’s** **Modulus** **(MPa)**		**UTS** **(MPa)**	**% Elongation** **at break**	**Young’s** **Modulus** **(MPa)**	
HPMC^17^	PEG	5.96	106	2.1		4.96	242	0.56	Oral fast dissolving film
Soluplus^16^	PEG	8.8	66.4	196.0		0.37	461	1.4	Drug release film
HPC^18^	PEG 400	7.83	51.42	—		6.99	55.91	—	Vaginal film
PMVE/MA^12^	Glycerol	4.73	14.72	1.14		2.02	36.54	0.04	Bioadhesive patch
Starch^19^	Glycerol	17.5	1.3	2120		1.2	5.6	26	Bioadhesive film
Cashew gum and CMC^20^	Glycerol	5.35	0.78	710		1.81	59	3	Biotechnology/packaging

Ref.^7^. Garg *et al*.^8^. Schilling *et al*.^9^. Limpongsa *et al*.^10^ Silva *et al*.^11^ Singh *et al*.^12^ Moss *et al*.^13^ Singh *et al*.^14^ Dana *et al*.^15^ Alanazi *et al*.^16^ Lim *et al*.^17^ Pandey *et al*.^18^ Dobaria *et al*.^19^ Rechia *et al*.^20^ Britto *et al*. Abbreviations used - UTS: ultimate tensile strength, PVA: poly vinyl alcohol, HPMC: hyrdoxy propyl methyl cellulose, PMVE/MA: (poly) methylvinyl ether/maleic anhydride, PVP: polyvinyl pyrrolidone, PEGDA: poly(ethylene glycol) diacrylate, PEG: polyethylene glycol, HPC: hydroxypropyl cellulose, EC: ethyl cellulose, PG: propylene glycol, TEC: triethyl citrate, PPG: polypropylene glycol, CMC: carboxy methyl cellulose.

**Figure 1:**
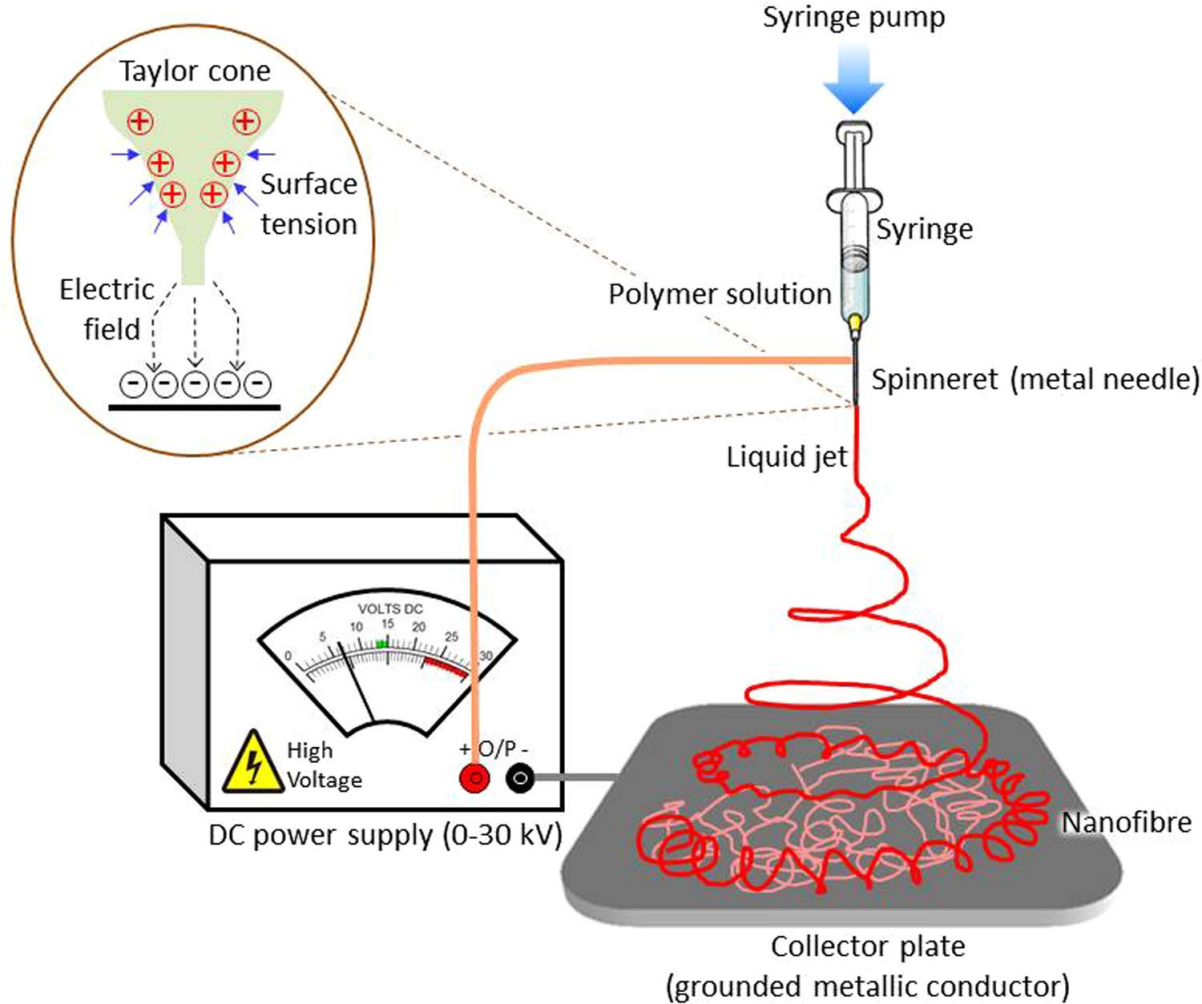
Electrospinning apparatus set-up and nanofiber mesh.

In this paper, for the first time we have investigated and reported potential of electro-spinning method (Fig. 1) over solvent casting method to fabricate polymeric membrane with enhanced flexibility and plasticity. We have systematically evaluated the phenomena by thorough investigation of electrospun and solvent casted membrane. We hypothesize that the mechanical properties and plasticity of electrospun polymer nanofibers will be influenced by the fabrication method. In this study, we employed scanning electron microscopy technology to evaluate the consequence of electrospinning method on morphology of spun fibers as well as on the morphology of solvent casted membrane. The change in mechanical properties and flexibility were also studied. How the fiber alignment itself change the mechanical properties of fiber, were studied. XRD, DSC were employed to support the potentiality of electrospinning process.

## Results

### Surface Morphology

**Figure 2:**
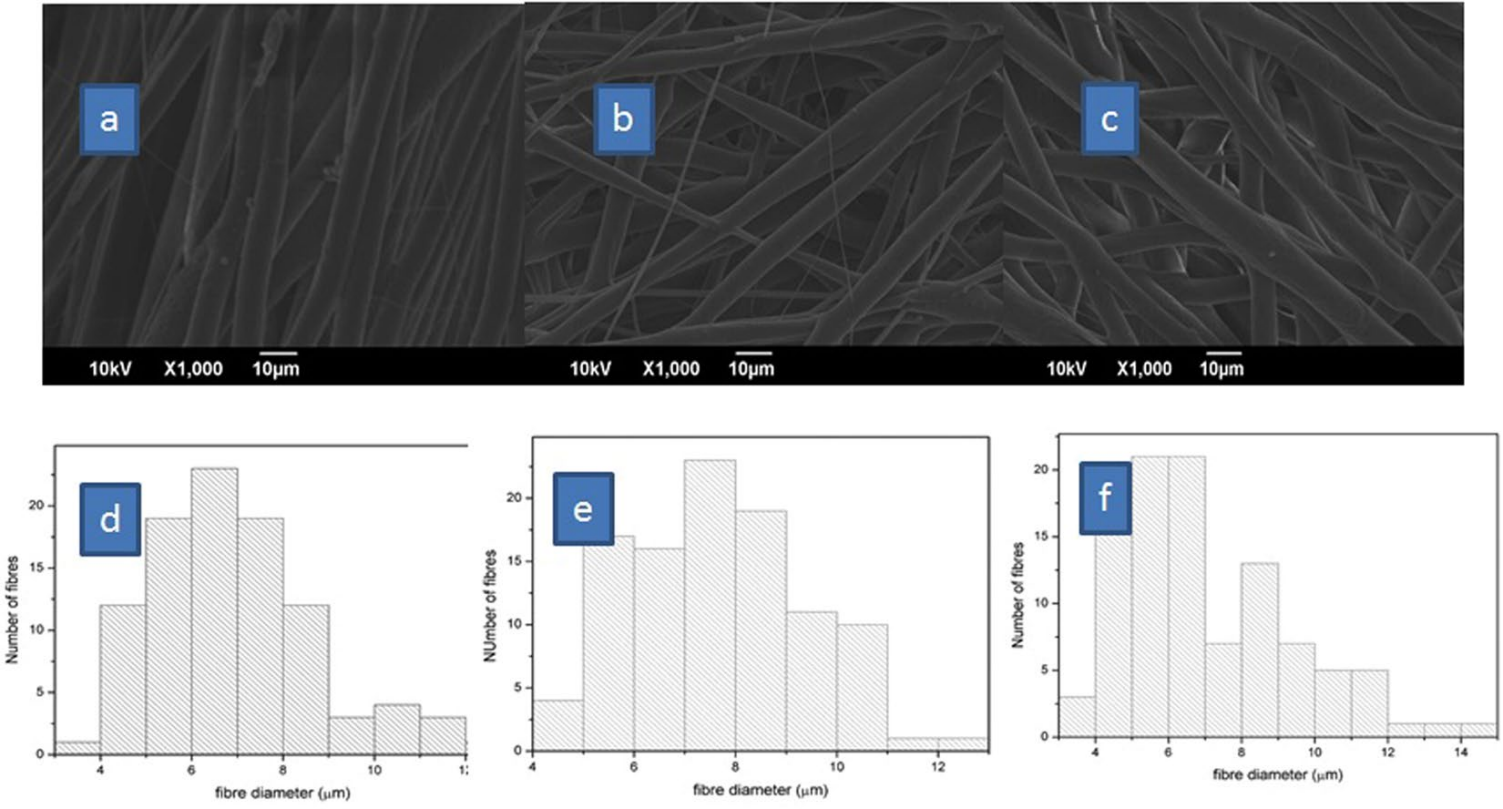
SEM images of electrospun membrane.

**Figure 3:**
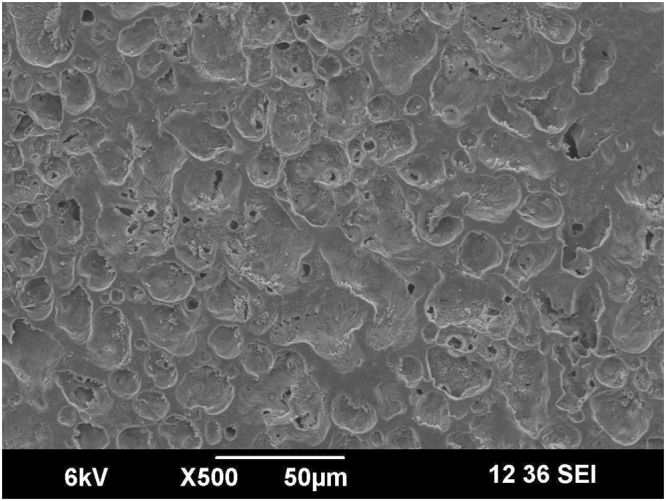
SEM images of solvent casted membrane.

Electrospinning is extensively used to develop fiber of high tensile strength, which is desirable in applications that need lightweight but durable materials. The ability to align and control fiber diameter makes this technique highly attractive. Here, we prepared three electrospun membranes with different kinds of alignment using a rotating mandrel. For simplicity, we designated membrane with uni-directionally aligned fibers as aligned, those randomly aligned as random, and those with aligned fibers overlaid on a randomly aligned as a bilayer. These differences tuned the morphology and size of the fiber because the aligned consisted of uniformly sized fibers that mainly ranged from 5–8 µm. In contrast, the random featured a mixed array of fibers in the range of 5–11 µm and the bilayer exhibited fiber diameter around 4–7 µm (Fig. 2). The SEM micrographs revealed the surface morphologies of the electrospun and the solvent-casted membranes (Figs 2 and 3). Evidently, electrospinning afforded membranes with aligned or non-aligned nano-sized fibers in contrast to that obtained from solvent-casting, which have an irregular surface morphology characterized by the absence of fibers and presence of crystals due to the slow evaporation process. Presumably, the nodules in the solvent-cast membranes are the crystallized domains.

### Mechanical characterization

**Figure 4:**
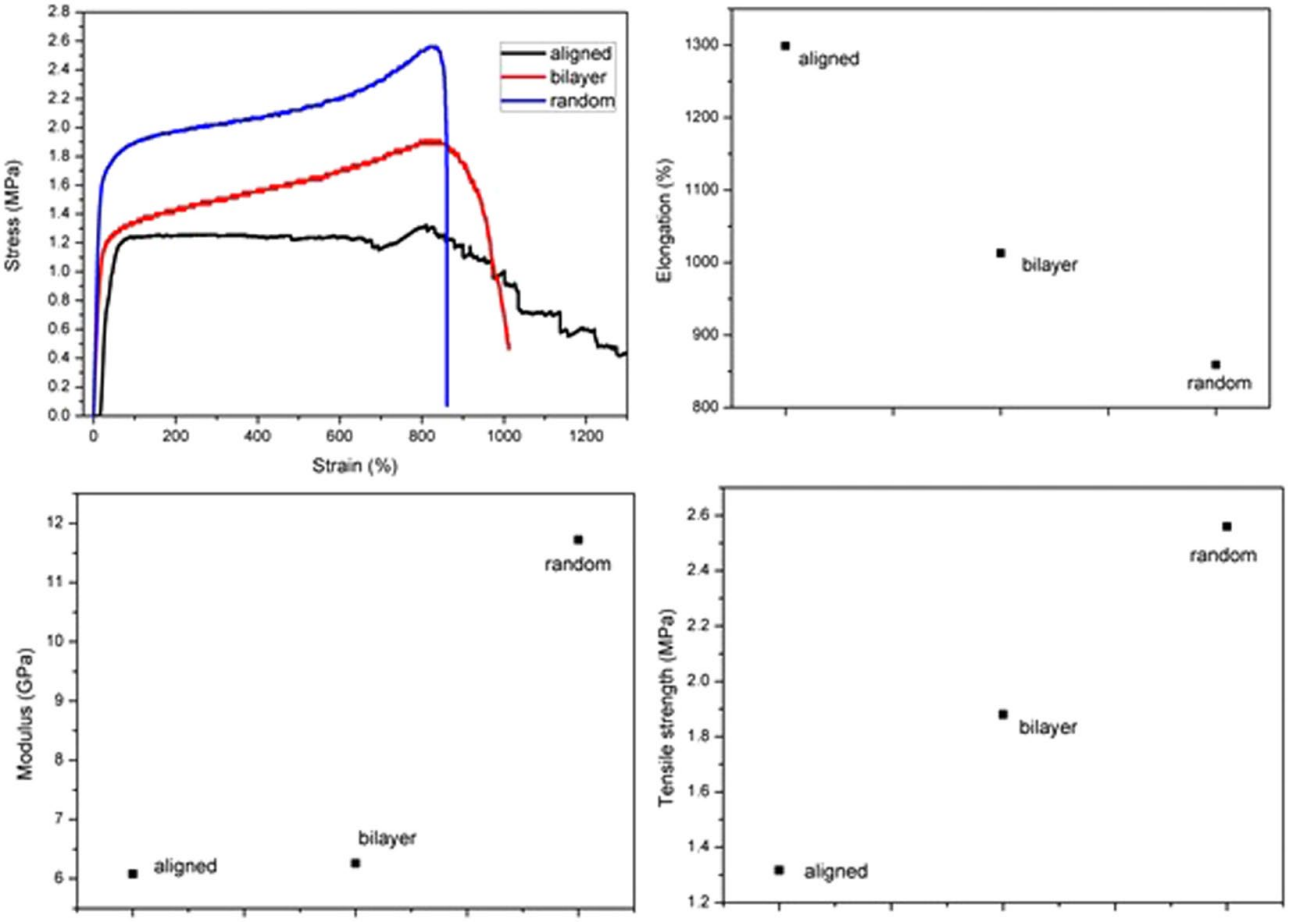
Mechanical properties of electrospun membrane.

Our investigations revealed that the electrospun PCL membranes excellent elongation rates with tensile strengths of 1.3, 2.6, and 1.9 MPa for aligned, random and bi-layered, respectively. The SEM micrographs of the random membrane reveal fusion among the fibers, a phenomenon that likely increases Young’s modulus and decreases elasticity. Fusion is lacking in the aligned and bilayer membranes, and should results in better orientation and stretching of fibers during mechanical stress. Indeed, the aligned and bilayer gave better elongation than the random (Fig. 4)^21–23^. We were unable to characterize the mechanical property of the solvent-cast membrane due to their brittleness.

### DSC study

**Figure 5:**
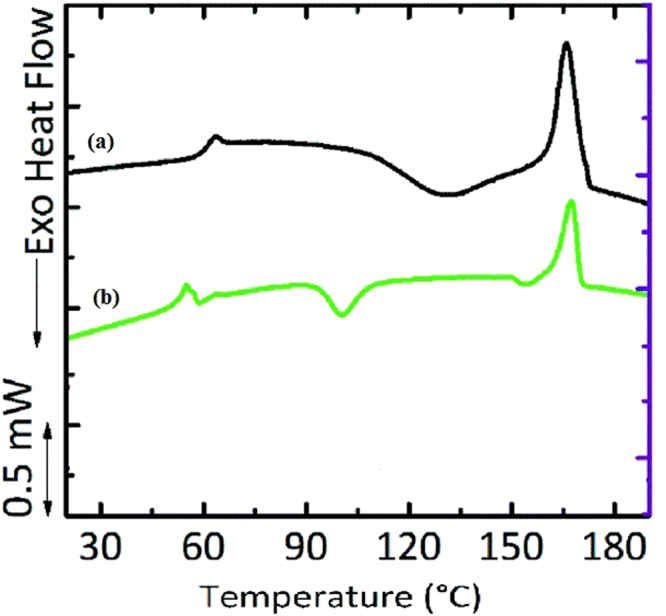
Representative DSC of (**a**) electrospun membrane (**b**) solvent casted membrane.

We employed DSC to probe the thermal-induced transitions in the electrospun and solvent-cast membranes. The solvent casting process involves slow solvent evaporation process, leading to the formation of crystals, which are not highly oriented. The thermal process during the DSC experiment partially realigned the crystalline domains as evidenced by the presence of a crystallization temperature at 100 °C (Fig. 5). In electrospinning process, the rapid solvent evaporation precludes crystal formation, and indeed, the DSC data reveal no crystallization peak.Regardless of the differences in the crystallization behaviors of these membranes, their melting temperatures were similar (~170 °C), but their enthalpy of melting (Hm) differed with the electrospun exhibiting higher enthalpy as evidenced by the higher endothermic peak. Perhaps higher heat energy is required to melt the highly oriented fiber in the electrospun membrane in contrast to the partially oriented crystalline domains in the solvent-cast membrane^24^.

### XRD study

**Figure 6:**
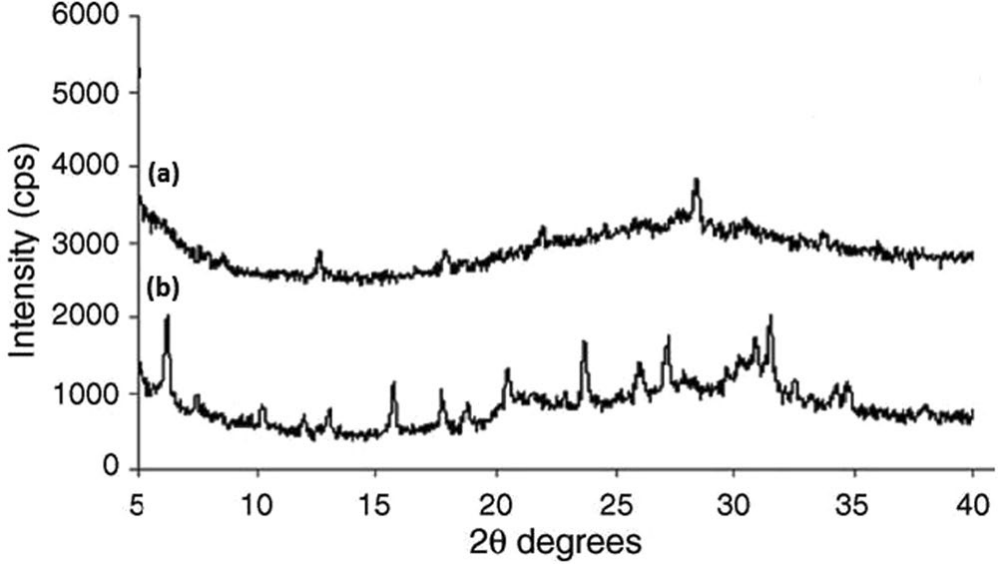
XRD study of (**a**) electrospun membrane (**b**) solvent casted membrane.

Powdered XRD experiments provide information on the degree of crystallinity in polymers. We conducted XRD experiments to gain insights into the degree of crystallinity in the membranes (Fig. 6). Apparently, the absence of crystalline reflections of PCL in the electrospun membrane suggests lack of crystalline domains. This contrasts the XRD diffractogram of the solvent-cast membrane, where sharp peaks were evident due to the presence of crystalline domains^25^.

## Discussions

In this present study, our goal includes understanding the fundamental difference in the mechanical behavior of differently oriented electrospun membranes, as well as the differences in surface morphology and thermal behavior of membranes fabricated via electrospinning and the popular solvent-cast. This knowledge is essential from the processing and practical application perspectives and provides parameters to tune mechanical, thermal and surface properties of membranes. To understand the mechanical properties, we characterized the ultimate tensile strength (UTS), Young’s modulus, and elongation at break (%EB). The orientation of the fiber within the electrospun membrane profoundly tunes UTS, %EB, and Young modulus. A random alignment yielded a membrane with higher UTS and Young modulus compared to orderly aligned fibers. The previous studies indicate that the mechanical properties of electrospun fibers depend on the direction of fiber alignment within the membrane. For instance, electrospun random membranes collected on static collectors have lower tensile strength than their aligned counterpart. In contrast, we observed that, with a rotating mandrel as the collector, the random membrane has higher tensile strength than the aligned. Also, in principle, electrospinning improves plasticity, forming flexible films. To the best of our knowledge, several researchers have enhanced plasticity using electrospinning, but a detailed mechanistic explanation is lacking. By comparing our amorphous electrospun to semi-crystalline solvent-cast membranes, we establish a link between the enhanced plasticity of electrospun with amorphousness. Our findings are very optimistic because it provides a viable alternative to overcome the common inflexibility problems of polymeric films prepared by solvent casting method without using plasticizers. Typically, plasticizers penetrate polymer networks reducing cohesive intermolecular forces among polymer chain, a phenomenon that reduces the tensile strength and increasing strain elongation and ultimately improves flexibility^26–28^.

**Figure 7:**
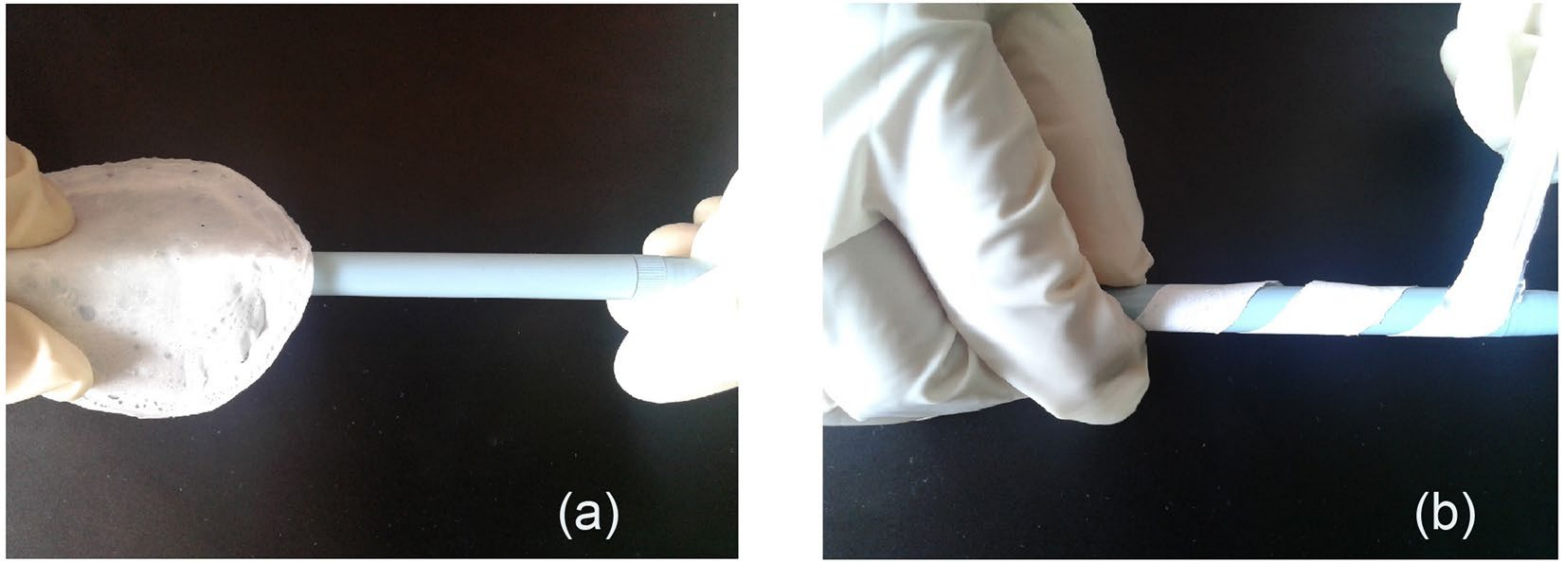
(**a**) Inflexible solvent-cast film and (**b**) flexible electrospun matrix of 12% w/v PCL solution.

Table 2 enlists some literature where it is notified that mechanical properties of the polymer films had been modulated or modified by many folds solely via electrospinning process^29–34^. Several process parameters tune the mechanical properties of the polymer films obtained via electrospinning process^29–34^. Very soft, smooth, and flexible but moderately strong thin polymer films are obtainable by electrospinning process^35,36^. To achieve this, investigators optimizes electrospinning parameters such as voltage, the distance between nozzle and collector, polymer concentration^37,38^. In our electrospinning experiment, we applied an electric voltage over polymer solution to draw nano-microfibers^39,40^ and deposit them as a non-woven matrix. Apparently, this non-woven matrix comprises of randomly aligned polymer chains entangled with each other with distinct voids or porosity (Fig. 3). The porosity between the polymer strands reduces cohesive force between polymer molecules. Also, these voids allow more space for the polymer chains to relax. Therefore, the polymer matrix will be easily strained under particular stress condition due to low cohesion and when the stress is removed polymer chains efficiently relax due to the presence of voids. In this study, the electrospun membranes were flexible in contrast to the membrane were cast from the same solvent blend and same polymer concentration but without plasticizer. We were unable to characterize the mechanical property of the solvent-cast membrane due to their brittleness. Indeed, Fig. 7 shows inflexibility of solvent-cast film and flexibility of electrospun matrix.

**Table 2:** Selected polymeric films obtained through electrospinning method.

Polymer	Findings	Application
CA^29^	Electrospun patches exhibit improved flexibility over the solvent cast films.	Transdermal patch
GE-GEM-GM^30^	Electrospun matrix showed viscoelastic behavior similar to blood vessels.	Small diameter vascular graft
PCL^31^	Electrospinning method produced strong but soft and flexible matrix like buttefly wing or human skin.	Tissue engineering
PU^32^	Large surface area and high porosity confirmed flexibility of the electrospun matrix in comparison to casted film.	Drug delivery
PVA^33^	Films made through solvent casting are brittle unless a high amount of glycerol is added. Through electrospinning method, strong and flexible films may be obtained even at very low concentration of glycerol.	Drug delivery
Zein^34^	Plasticizers used in solvent casting method affect barrier property of zein film negatively. Electrospinning produces flexible films without affecting barrier properties.	Food packaging

Ref.^29^. Taepaiboon *et al*.^30^ Thomas *et al*.^31^ Bölgen *et al*.^32^ Saha *et al*.^33^ Tyagi *et al*.^34^ Fabra *et al*. Abbreviations used - CA: cellulose acetate, GE-GEM-GM: gelatin/elastin, gelatin/elastin/maxon, and gelatin/maxon, PCL: poly(ε-caprolactone), PU: poly urethane.

## Conclusions

The mechanical properties of polymer films determine their processing properties and applications. Electrospinning enhances the plasticity of polymer films and membranes, improving flexibility without compromising durability. Here, we establish a link between the enhanced plasticity of an electrospun membrane to amorphousness. Indeed, membranes cast from the same solvent and featuring semi-crystalline domains were brittle and inflexible in contrast to amorphous electrospun membranes. Also, the orientation of the fibers within the membrane tuned the mechanical properties.

## Materials and Methods

### Materials

poly(ε-caprolactone) (Mw 80,000) was obtained from Sigma Aldrich, USA while analytical grade chloroform was obtained from SRL, Mumbai.

### Sample preparation

To formulate the casting and electrospinning solution, we dissolved poly(ε-caprolactone) in chloroform at a concentration of 12% w/v and ensured homogeneity by agitation. The procedure is as follow: an appropriate amount of the polymer was added to the required amount of solvent and stirred for 2 hrs. Then, the resulting mixture was sonicated for 15 minutes and again stirred for another 12 hrs. This solution was used to fabricate the nanofibrous polymer matrix as well as the solvent-cast polymer films. Before electrospinning and casting, the solution was sonicated for 15 minutes and was allowed to sit for 30 minutes to de-aerate.

### Electrospinning method

The electrospinning process involves the use of a high DC voltage of several kV (10–20 kV) to induce electrical charges on a jet of polymer solution which dries leaving a polymer nanofiber, eventually forming a nanofibrous mat. The experimental setup includes an electrode connected to the spinneret or metal needle of the syringe that holds the spinning solution, another electrode connected to an 11 × 7 cm aluminum sheet collector, and a syringe pump that drives the polymer solution through the syringe (Fig. 1). To carried out the electrospining, we transferred the polymer solution to a 10-mL syringe fitted with a 0.7 mm gauge blunt needle tip, and electrospun (Holmark, Model- HO- NEV-02) at varying voltages (10 kV to 20 kV) while maintaining the flow rate at 1 mL/hr. The electrospinning was carried out using a rotating mandrel at different speeds (300 to 1000 rpm). The process yielded three kinds of membranes, aligned, random, and bilayer (aligned fibers deposited on random mats), which were used for further studies.

### Solvent casting method

The polymer solution was transferred to a Petri dish, covered with an inverted funnel, and allowed to sit overnight to dry. After drying, the sample was collected and stored in desiccators until further use.

### SEM study

Electrospun mat or the solvent-casted film sectioned into specific length and width were coated with a platinum layer using a JEOL JFC 1600 Auto fine coater. The images were obtained at 20 kV and then analyzed by software Digimizer® in order to calculate the fiber diameter.

### Mechanical characterization

Following ASTM D 882 standard, we characterized the tensile strength of the electrospun nanofibers using Universal Tensile Machine (UTM) INSTRON 1408. Tensile testing was carried out using 500 N load cells at a speed of 1 mm/min onto the specimen. The samples were prepared with a width of 5 mm, a gauge length of 20 mm, and a thickness of 0.2 mm. We performed these experiments in triplicate.

### Differential scanning calorimetry

Thermal analysis was performed by DSC (Pyris Diamond TG/DTA; Perkins Elmer Instruments, Mumbai, India). Samples (about 5–10 mg) were heated from 25 to 200 °C at a heating rate 10 °C/min under a steady stream of inert nitrogen gas (flow rate 150 ml/min).

### Powdered XRD spectroscopy

The X-ray measurements of nanofibers and solvent-casted membrane were carried out using a Bruker D8 ADVANCE X-ray diffractometer with a scanning rate of 2° per min using Cu–Kα radiation. The XRD data were collected in the 2θ range of 5–60°.

### Data availability statement

The datasets generated during and/or analysed during the current study are available from the corresponding author on reasonable request.
